# Phylogenetic insights into the diversification of cutting strategies in leaf-cutting ants

**DOI:** 10.3389/finsc.2026.1778418

**Published:** 2026-04-07

**Authors:** Andrés F. Sánchez–Restrepo, Viviana A. Confalonieri, Luis A. Calcaterra

**Affiliations:** 1 Fundación para el Estudio de Especies Invasivas (FuEDEI), Hurlingham, Buenos Aires, Argentina; 2 Instituto de Ecología, Genética y Evolución (IEGEBA), Consejo Nacional de Investigaciones Científicas y Técnicas (CONICET)-Universidad de Buenos Aires, Buenos Aires, Argentina; 3 Facultad de Ciencias Exactas y Naturales, Universidad de Buenos Aires (UBA), Buenos Aires, Argentina; 4 Departamento de Biodiversidad y Biología Experimental, Instituto de Biodiversidad y Biología Experimental y Aplicada (IBBEA), Consejo Nacional de Investigaciones Científicas y Técnicas-Universidad de Buenos Aires, Buenos Aires, Argentina

**Keywords:** ancestral state reconstruction, convergent evolution, divergence time estimation, evolutionary diversification, foraging specialization, Miocene-Pliocene, neotropical ecosystems

## Abstract

Leaf-cutting ants are dominant herbivores in Neotropical ecosystems, yet the evolutionary origins of their cutting preferences remains unresolved. We investigated whether grass-cutting specialization emerged from a single evolutionary innovation or multiple independent origins. We compiled the predominant cutting behavior of each leaf-cutting ant species and classified them as dicot, grass, or grass–dicot cutters. Integrating these data into a time-calibrated phylogenetic framework allowed us to reconstruct the evolutionary history and diversification of leaf-cutting behavior in these ants. Divergence-time analyses date the origin of leaf-cutting ants to the early Miocene, major crown clades diversified during the late Miocene to early Pliocene, a period of increasing climatic seasonality and landscape openness in South America. The evolutionary history of these ants is strongly influenced by large-scale climatic and geological processes, as evidenced by their origin and major diversification events in close association with Miocene–Pliocene environmental changes. Phylogenetic analyses clarify the contrasting diversification dynamics of *Acromyrmex*, *Amoimyrmex* and *Atta*. *Acromyrmex* traces back to ~15 Ma and is structured into at least four major clades, with a distinct grass cutting clade. In contrast, *Atta* is a younger lineage characterized by rapid diversification during the Pliocene. *Amoimyrmex* occupies an early diverging position, indicating an independent origin of grass cutting within the group. Ancestral state reconstructions consistently indicate that dicot cutting is the ancestral condition, with grass and mixed dicot–grass cutting evolving independently multiple times. These repeated transitions coincide with the temporal expansion of open, grass-dominated habitats and likely reflect adaptive responses to new ecological opportunities. The convergent evolution of grass-cutting strategies, despite the mechanical and ecological challenges posed by silica-rich grasses, suggests that cutting preferences are evolutionarily flexible yet functionally constrained traits. Together, our results suggest that grass-cutting is not phylogenetically conserved, but rather represents a recurring adaptive response to environmental change. This highlights how major landscape transformations during the Miocene–Pliocene period promoted repeated ecological innovation in socially complex herbivores.

## 1 Introduction

The evolution of dietary preferences and specialization represents a pervasive phenomenon across animals and plants, that is often traceable through phylogenetic patterns of convergence, constraint and ecological opportunity. Comparative phylogenetic studies have shown that dietary traits often evolve in bursts, following environmental or geological transitions that alter resource availability and plant community composition (1–3). Studies across different taxa have shown that distinct feeding strategies evolve repeatedly in lineages, accompanied by shifts in sensory and morphological traits, such as mandible or jaw structure, and chemosensory gene repertoires (4). These transitions often coincide with eco-geological events, such as climate-driven changes in vegetation or biotic radiations, which create new ecological niches (4, 5). Phylogenetic analyses suggest that, depending on the timing and magnitude of ecological opportunity, diet specialization can constrain or promote diversification (6, 7).

Leaf-cutting ants (genus *Acromyrmex*, *Amoimyrmex*, and *Atta*) are fascinating eusocial insects renowned for their complex behaviors and unique ecological role as dominant herbivores in Neotropical ecosystems. They form a well-defined monophyletic group and are the most derived members of the fungus growing ants, also known as Attini group (8, 9). The great innovation that made leaf-cutting ants one of the dominant herbivores in the Neotropics stems from the evolutionary innovation of cutting fresh plant material, such as leaves, flowers, and grasses to cultivate a symbiotic fungi (10). Their success is further facilitated by their ability to inhabit a wide variety of climates with different types of vegetation (11); by nest structures that maintain optimal microclimatic conditions for the growth of their fungal symbionts (12, 13); and by their hygiene habits, which prevent infections and protect crops from parasites (14).

The morphological and behavioral differences between leaf-cutting ant genera emphasize their evolutionary and ecological diversity. These genera differ in terms of the arrangement and presence of mesosoma spines, the presence or absence of tubercles on the gaster, the degree of caste polymorphism and colony size (15). Leaf-cutting ants exhibit distinct cutting preferences, determining which plant species and tissues are harvested and supplied to their cultivated fungal symbionts. These preferences are not uniform across species, but rather reflect specialized ecological adaptations. While most leaf-cutting ants predominantly harvest dicot leaves, some species clearly and consistently specialize in harvesting grasses, while others switch between grasses and dicots depending on local resource availability (16, 17). These preferences influence not only their foraging strategies, but also broader ecological traits such as nest-site selection and habitat use, thereby demonstrating the adaptive diversity within leaf-cutting ants. Leaf mechanical traits, particularly leaf toughness and age, consistently influence worker choice and cutting rates, making plant physical properties key proximate drivers of substrate selection by these ants (18). At the symbiotic level, the ant’s fungal cultivar produces different sets of biomass-degrading enzymes when fed grasses versus dicots. This indicates that the enzymes respond specifically to different substrates, facilitating the extraction of nutrients from distinct plant tissues (19). Similarly, metagenomic studies have revealed differences in bacterial community composition linked to diet between grass- and dicot-cutting colonies, suggesting that the entire ant-fungus-microbiome complex is adapted to the type of substrate (17). Within *Acromyrmex*, ecological and behavioral adaptations for grass utilization (such as nest turrets that incorporate grass fragments) demonstrate that monocot specialization is not exclusive to the genus *Atta* (20). Historically, *Acromyrmex* has been divided into two subgenera, *Moellerius* and *Acromyrmex*, based on morphological characters (21–23). Subgenus *Moellerius* species are characterized by short, robust mandibles and the absence of supraocular spines, traits associated with cutting grasses. In contrast, the subgenus *Acromyrmex* includes species with longer mandibles and supraocular spines, which are primarily adapted to cutting dicots. However, different phylogenetic studies have shown evidence challenging the validity of this division and shown that species like *Acromyrmex* (*Moellerius*) *heyeri* and *Acromyrmex* (*Acromyrmex*) *lundii* are closely related (24, 25).

Cutting preferences arise from an interplay of selective forces acting at multiple biological levels. From the plant’s side, mechanical toughness and chemical defenses influence the cost and risk of harvesting specific tissues. Ant sensory systems and learning abilities enable them to distinguish and respond to plant cues (26–28). On the ant side, mandible morphology and bite force, both of which scale with worker size, place mechanical limits on which tissues can be efficiently cut (29, 30). Colony-level traits, such as worker size distribution and division of labor, also influence actual foraging behaviors because polymorphic colonies can assign specialized workers to various cutting tasks (31). Behavioral and developmental plasticity enable colonies to adapt their cutting behavior to heterogeneous environments and may facilitate evolutionary shifts in preference (32).

In this study we aim to investigate the evolutionary origins and diversification of cutting preferences in leaf-cutting ants using a phylogenetic framework. By integrating behavioral information with evolutionary relationships, we will try to determine if grass- and dicot-cutting species diverged along distinct evolutionary trajectories or if they are examples of parallel adaptations to historical changes in plant communities. Specifically, we examine how ecological pressures, niche differentiation, and long-term environmental dynamics have shaped the evolution of foraging specialization within the leaf-cutting ants. Through this approach, we will assess whether transitions in cutting behavior correspond to key events, as well as to broader patterns in the natural biogeographic history of the American continent.

## 2 Materials and methods

### 2.1 Cutting preferences in leaf-cutting ants

We compiled cutting preferences for each leaf-cutting ant species from published literature and our field observations. For all species, we recorded whether species were classified as predominantly i) dicot, iii) grass, or iii) grass-dicot cutting based on relative harvest frequencies under natural conditions, according to the reviewed literature (20, 33–36) and our own field observations. We employed a hierarchical approach to this categorization, aiming to capture dominant foraging behavior rather than strict exclusivity. For species with mixed diets, where the predominant preference could not be clearly determined from behavioral data alone, mandible morphology was used as complementary evidence rather than as the primary basis for classification. In these cases, morphological traits were only considered to support or clarify patterns suggested by literature and field observations. For instance, when a species exhibited a predominantly dicot diet with occasional grass harvesting, we evaluated whether the mandible structure was consistent with functional specialization. Specifically, we considered distinct morphological traits reported for grass-cutting species, such as shorter, more robust mandibles optimized for processing tough, narrow leaves, to reinforce an observed tendency towards grass use (37). Conversely, sharper and more dentate mandibles suited to shearing broadleaf tissue were interpreted as consistent with a predominant tendency to cut dicots. Furthermore, we cross-referenced these functional traits with the known distribution of each species across American biomes (following 38; see **Supplementary Material**) to align cutting preferences with the dominant vegetation of their respective habitats. Together, integrating behavioral data and ecological distribution enabled us to consistently characterize cutting preference tendencies across species for subsequent phylogenetic analyses.

### 2.2 Collection and processing of genetic data

We extracted genomic DNA from one worker per sample using a REDExtract-N-Amp kit (Sigma-Aldrich, USA). Five fragments were amplified by PCR: one mitochondrial region (COI–IGS–tRNA-Leu–COII) and four nuclear genes (EFαF1, EFαF2, Wg, and LWRh). Detailed PCR conditions and primer sequences are provided in the **Supplementary Material**. Then, the purified PCR products were sequenced using an ABI 3130XL Genetic Analyzer (Applied Biosystems) at the Sequencing and Genotyping Unit of the University of Buenos Aires (FCEyN, UBA, Buenos Aires, Argentina) and at Macrogen Inc. (Korea). Sequences were inspected, trimmed, and aligned in Geneious Pro v4.8 (http://www.geneious.com/) using the MUSCLE algorithm (39). We verified that coding regions lacked gaps altering the reading frame. Intergenic regions, introns (e.g., in LWRh) were removed. In particular, for the COI-IGS-tRNAleu-COII region, the position of the tRNAleu was verified using the tRNAscan-SE online server (40) and the intergenic spacer was excluded due to alignment unreliability.

### 2.3 Taxon sampling

We generated new mitochondrial and nuclear sequence data for as many species as possible, supplementing with publicly available sequences. Samples for new sequences were collected during field expeditions in southern South America (primarily Argentina) from 2015 to 2020. In total, the genetic dataset includes 21 species of *Acromyrmex*, 11 species of *Atta*, and 3 species of *Amoimyrmex*. To provide broader phylogenetic context, we also included outgroup sequences from closely related genera of higher fungus-growing ants (*Trachymyrmex* sensu stricto, *Paratrachymyrmex*, *Mycetomoellerius*, *Sericomyrmex*, and *Xerolitor*), representatives of the major groups of other fungus-growing ants (*Mycetagroicus*, *Mycetophylax*, *Cyphomyrmex*, *Mycetarotes*, *Mycetosoritis*, *Kalathomyrmex*, *Cyatta*, *Apterostigma*, *Myrmicocrypta*, and *Mycocepurus*), and more distantly related genera (*Acanthognathus*, *Blepharidatta*, *Cephalotes*, *Daceton*, *Ochetomyrmex*, *Monomorium*, *Pheidole*, *Procryptocerus*, and *Wasmannia*).

### 2.4 Phylogenetic reconstruction and dating

Accurate estimation of clade ages in a phylogenetic framework requires external calibration, commonly based on fossil constraints or independently derived nucleotide substitution rates. One challenge in estimating the age of leaf-cutting ants is the absence of fossils. The closest fossil evidence we have is a trace fossil (ichnotaxon) from a Miocene nest in Argentina. Laza (41) described it as †*Attaichnus kuenzelii* and attributed it to ants of the *Acromyrmex* or *Trachymyrmex* s.l. genera (42). However, there are known fossils of fungus-growing ants, such as *Apterostigma*, *Cyphomyrmex*, and *Trachymyrmex* s.s., that can be used as calibration points due to their phylogenetic closeness to leaf-cutting ants. Thus, for the divergence calibration we include six fossils within the outgroup: three from fungus-growing ants and three from more distant outgroup species (**Supplementary Table 2**).

We used a Bayesian approach in BEAST v. 2.5.1 (43) to perform the phylogenetic analysis, calibration, and age estimation. This approach used the “fossilized birth-death process” (FBD) model, which is considered appropriate for calibrating divergence time estimates (44). Unlike a total-evidence analysis, the FBD model does not require a morphological matrix of fossils; rather, it assigns fossils to clades based on topological constraints (45). In other words, the observed fossils are treated as part of the prior of temporal nodes, which includes them in the process that governs tree topology and branch times (44). Substitution models were unlinked for the nuclear data and linked for the mitochondrial data, and were estimated using bModelTest package (46). This package allows one to explore the parameter space of the surrogate model while simultaneously estimating model parameters and phylogeny. The root age was set to 98.6 million years ago (Ma), according to the mean age estimated for the subfamily Myrmicinae (47). Tree parameters were linked across partitions, and an uncorrelated relaxed clock model (48) was used. The analysis was performed using two independent Markov chain Monte Carlo (MCMC) replicates, each with 60 million generations and sampled every 1,000 generations. Convergence was assessed in Tracer v1.6 (43) by ensuring that the effective sample size (ESS) for each estimated parameter was greater than 200. Both replicates were combined in LogCombiner, applying a burn-in of 25% for each run. Fossils were removed from the resulting trees using the “FullToExtantTreeConverter” tool in Beauti v2.5. Then, a maximum credibility tree was generated in TreeAnnotator (43). Node ages were validated against published fungus-growing ant phylogenies (8, 49, 50).

### 2.5 Reconstruction of ancestral cutting preferences

To test phylogenetic associations with the previous compiled cutting preferences (grass-, dicot-, dicot-grass cutting), we performed ancestral state reconstruction on the calibrated tree. We used to optimize the ancestral states of the cutting habit type at the nodes with the “rerootingMethod” function of the R “phytools” package, version 0.7-12 (51), which uses Yang et al. (52) method. This optimization uses a re-rooting to estimate marginal ancestral states using likelihood. The model was fitted with equal rates of change. Internally, the function uses the Mk model for discrete trait evolution (51). Additionally, to characterize the temporal acquisition of feeding preferences, we performed stochastic character mapping. We simulated 100 stochastic realizations of trait evolution under an equal rates model (53). For each simulation, we extracted the absolute timing of transitions into specific character states and binned them across a 200-point time grid spanning from the root to the present. Then, we calculated the mean cumulative number of lineages possessing each trait at every time interval to generate an accumulation plot. This allowed us to make a direct comparison between the diversification of crown clades and the origin of the “grass-cutting” behavior.

## 3 Results

### 3.1 Cutting preferences in leaf-cutting ants

The cutting preferences of most leaf-cutting ants were documented (**Table 1**). Of the 46 valid species, 27 were identified as dicot cutters, including 18 *Acromyrmex* species and nine *Atta* species. Grass-cutting was recorded for 12 species, including five *Acromyrmex*, three *Amoimyrmex*, and four *Atta* species. Four species exhibited dicot-grass cutting behavior (two *Acromyrmex* and two *Atta*). There was no documented information on cutting preferences for *Acromyrmex evenkul* and *Acromyrmex pubescens*. Of these species we can include a total of 34 species in the subsequent phylogenetic analyses.

**Table 1:** Compiled cutting preferences (grass- vs. dicot-cutting) for the taxonomic valid known leaf-cutting ant species, along with the corresponding literature references.

Genus	Species	Cutting preference	In phylogeny	Reference
*Acromyrmex*	*ambiguus*	Dicot	Included	1,2,3,8
	*ameliae*	?	Included	
	*aspersus*	Dicot	Included	2,3
	*balzani*	Grass	Included	1,2,4,3,5
	*coronatus*	Dicot	Included	1,2,3,4
	*crassispinus*	Dicot	Included	2,3,8
	*diasi*	Dicot?-Grass		2,3
	*disciger*	Dicot	Included	1,2,3
	*evenkul*	?		
	*fracticornis*	Grass	Included	1,3,5,8
	*heyeri*	Grass	Included	2,3,8
	*hispidus*	Dicot	Included	1,2,3,8
	*hystrix*	Dicot		1,2,3,
	*landolti*	Grass	Included	1,2,3,4,5,8
	*laticeps*	Dicot	Included	1,2,3,8
	*lobicornis*	Dicot-Grass	Included	2,3,6,8
	*lundii*	Dicot	Included	1,2,3,8
	*molestans*	Dicot?		
	*niger*	Dicot	Included	1,2,3
	*nigrosetosus*	Dicot	Included	1
	*nobilis*	Dicot		2,3
	*octospinosus*	Dicot	Included	1,2,3,4
	*pubescens*	?		
	*pulvereus*	Grass		2,3
	*rugosus*	Dicot	Included	1,2,3,4
	*santschii*	Dicot?		
	*subterraneus*	Dicot	Included	1,2,3,8
	*versicolor*	Dicot	Included	2,3
*Amoimyrmex*	*bruchi*	Grass?	Included	
	*silvestrii*	Grass	Included	2,3,8
	*striatus*	Grass	Included	2,3,8
*Atta*	*bisphaerica*	Grass	Included	2,3,4
	*capiguara*	Grass		2,3,4
	*cephalotes*	Dicot	Included	2,3,4,8
	*colombica*	Dicot	Included	2,3,4
	*cubana*	Dicot?		
	*goiana*	Grass		2,3
	*insularis*	Dicot		2,3,4
	*laevigata*	Dicot-Grass	Included	2,3,4
	*mexicana*	Dicot-Grass	Included	2,3,4
	*opaciceps*	Dicot	Included	2,3
	*robusta*	Dicot	Included	2,3
	*saltensis*	Dicot	Included	3,8
	*sexdens*	Dicot	Included	2,3,4,8
	*texana*	Dicot	Included	2,3,4
	*vollenweideri*	Grass	Included	2,3,4,7,8

The question mark (?) in the cutting preferences indicate that the preference is not well documented. *A. ameliae* is known to be a social parasite, is included in phylogeny but can’t be defined as specific cutting preference. References follow 1: 33, 2: 34, 3: 36, 4: 35, 5: 20, 6: 54, 7: 55, 8: Authors field observations.

**Figure 1:**
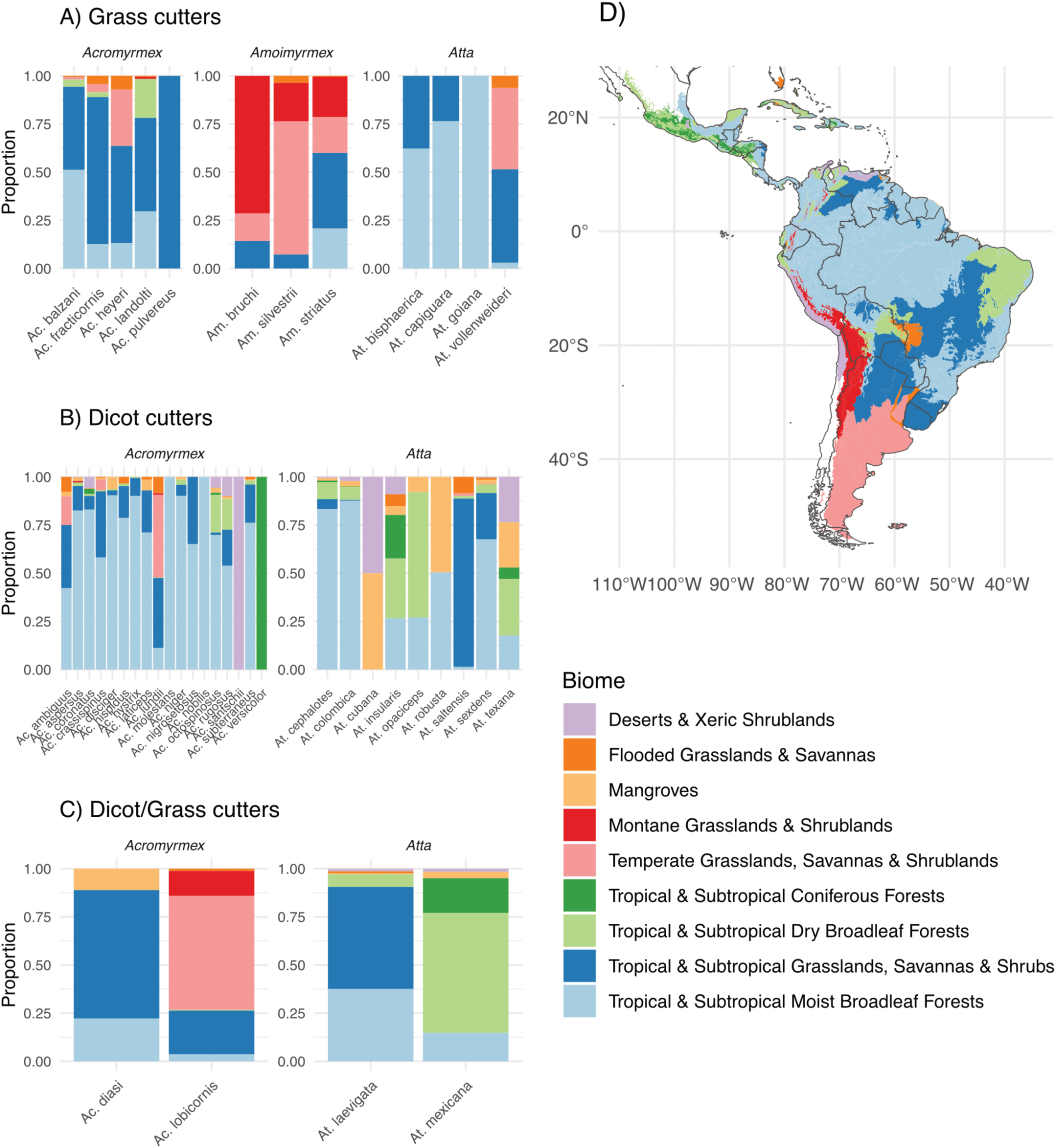
Biome-level distribution of cutting preferences in leaf-cutting ants. The left side shows panels corresponding to **(A)** grass cutters, **(B)** dicot cutters, **(C)** dicot-grass cutters, and **(D)** a map that shows the geographic extent of each biome. Within each barplot panel, the bars represent the proportion of occurrence records for each species across biomes. The colors on the map correspond to those in the bar plots.

The geographic distribution of cutting preferences was examined in relation to biome distribution (**Figure 1**). The grass-cutting species were primarily distributed across Tropical & Subtropical Grasslands, Savannas & Shrublands, Tropical & Subtropical Moist Broadleaf Forests, and Temperate Grasslands, Savannas & Shrublands. *Amoimyrmex* had a more geographically constrained distribution, occurring mainly in Temperate Grasslands, Savannas & Shrublands and Montane Grasslands & Shrublands. Dicot-cutting species were predominantly associated with Tropical & Subtropical Grasslands, Savannas & Shrublands and Tropical & Subtropical Moist Broadleaf Forests, but occupied a broader range of biomes overall; notably, *Atta* species showed a substantial presence in Tropical & Subtropical Dry Broadleaf Forests. In contrast, species exhibiting grass- and dicot-cutting behavior did not show consistent biome-level patterns; their distributions appeared to be species-specific.

### 3.2 Phylogenetic analyses

We successfully reconstructed and dated a phylogenetic tree for leaf-cutting ants using combined nuclear (EFαF1, EFαF2, Wg, LWRh) and mitochondrial (COI–tRNAleu–COII) markers, including species representing all three cutting categories (grass, dicot, and dicot-grass cutters). Divergence times were estimated under a birth–death fossilized model, and the final dataset comprised 33 of the 50 described leaf-cutting species: 21 *Acromyrmex*, 10 *Atta*, and 2 *Amoimyrmex*. These results provide a well-resolved temporal framework for examining evolutionary patterns across cutting preference categories. While taxonomic sampling is incomplete, the tree includes two-thirds of all currently described leaf-cutting ant species, supporting the robustness and reliability of the inferred divergence patterns.

**Figure 2:**
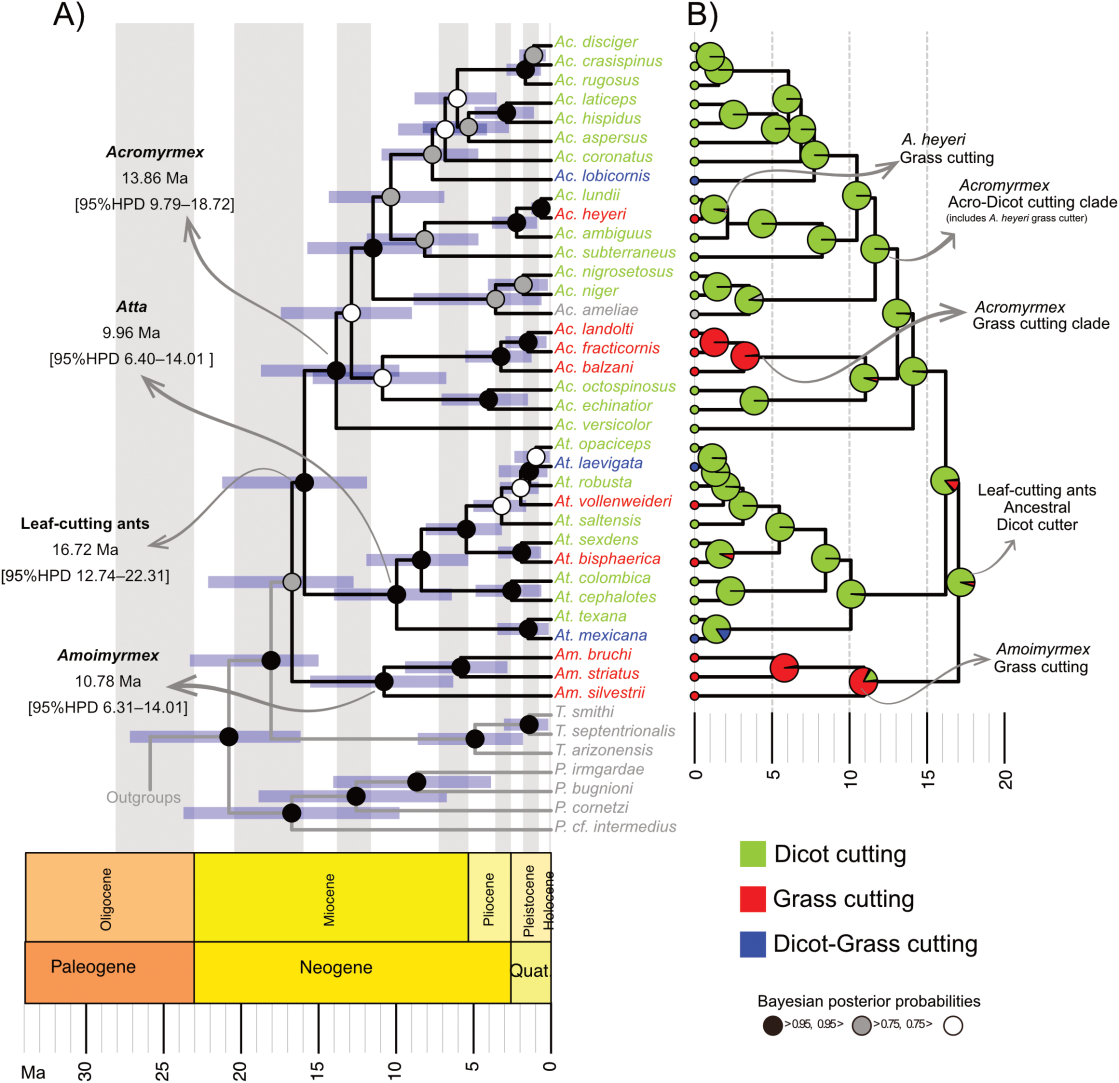
Time-calibrated phylogeny and ancestral state reconstruction of cutting preferences in leaf-cutting ants. **(A)** Maximum clade credibility tree obtained from Bayesian divergence-time analyses using the fossilized birth-death process model. The blue bars at the nodes indicate the 95% highest posterior density (HPD) intervals for the ages of the nodes, and the colors of the nodes represent the Bayesian posterior probabilities. **(B)** The same phylogeny, annotated with ancestral state reconstruction of cutting preference. The pie charts at each node show the posterior probability of each of the character states: dicot cutting (green), grass cutting (red), dicot-grass cutting (blue), and social parasite (grey). The geological time scale is shown at the bottom of both panels.

Bayesian divergence-time analyses based on the fossilized birth–death process produced a well-resolved, time-calibrated phylogeny of leaf-cutting ants and related attine taxa (**Figure 2A**). These results indicate that leaf-cutting ants originated in the mid-Miocene era, approximately 16.72 Ma (95% highest posterior density [HPD]: 12.745–22.131 Ma). Within the group, *Amoimyrmex* originated ~10.78 Ma (95% HPD: 6.31-15.547) and was identified as a sister taxon to a clade containing *Atta* and *Acromyrmex*. The maximum clade credibility tree shows that the diversification of the focal groups occurred primarily during the late Miocene to Pliocene period, with most major lineages arising within ~10 million years ago (Ma).

The genus *Acromyrmex* was divided into at least four major clades and appeared ~13 Ma (95% [HPD]: 9.79-18.724 Ma). Three of them diverged around 10 million years ago and one of these lineages corresponds to a distinct clade of only grass cutting species, which originated more recently at around 4 Ma. The temporal placement of this split suggests that grass cutting originated within *Acromyrmex* in the late Miocene-early Pliocene era. These clades exhibit relatively deep internal divergences compared to those observed in *Atta*.

The genus *Atta* originated ~10 Ma (95% [HPD]: 6.4-14.01 Ma) Unlike *Acromyrmex*, it exhibited more recent and rapid diversification. The dated phylogeny revealed four major clades within *Atta*, all of which emerged around 5 Ma or later. The divergence times of these clades overlap, suggesting a relatively narrow temporal window of radiation during the Pliocene. The comparatively short internal branches within *Atta* are consistent with a pattern of recent and rapid lineage splitting.

### 3.3 Ancestral state of cutting preferences

Ancestral state reconstruction of cutting habits (**Figure 2B**) consistently identified dicot cutting as the ancestral condition for leaf-cutting ants. Marginal posterior probabilities for deep nodes in the phylogeny strongly supported this state (P[dicot] ≥ 0.83 across basal nodes), indicating a high level of confidence in this reconstruction and providing negligible support for alternative states. Grass cutters and dicot-grass cutters were inferred as derived conditions that arose multiple times independently. Several internal nodes showed a clear shift towards grass cutting, with high posterior probabilities for this state (e.g., nodes 55–56, where P[grass]≥0.98, and nodes 68–69, where P[grass]≥0.81), which supports at least four independent origins of grass cutting across the dated phylogeny. The dicot-grass cutters were also reconstructed as a derived condition, appearing sporadically and without strong phylogenetic clustering.

Within the genus *Acromyrmex*, a well-supported, monophyletic only grass cutting clade comprising the species *A. landoltii*, *A. fracticornis* and *A. balzani*. This clade originated ~4 Ma and is characterized by a high posterior probability of grass cutting at its ancestral node (**Figure 2B**). By contrast, the remaining *Acromyrmex* species formed a predominantly dicot-cutting clade, with the notable exception of *A. heyeri*. This species was recovered as the sister species to *A. lundii* and showed evidence of a derived cutting preference.

In *Atta*, the reconstruction of ancestral states did not identify distinct clades corresponding to grass cutters or dicot-grass cutters. Instead, species exhibiting these cutting habits were scattered throughout the genus, and the inferred ancestral nodes retained strong support for dicot cutting. This pattern suggests that shifts towards grass or dicot-grass cutting preferences in Atta occurred independently in multiple species rather than through the diversification of a single specialized clade.

**Figure 3:**
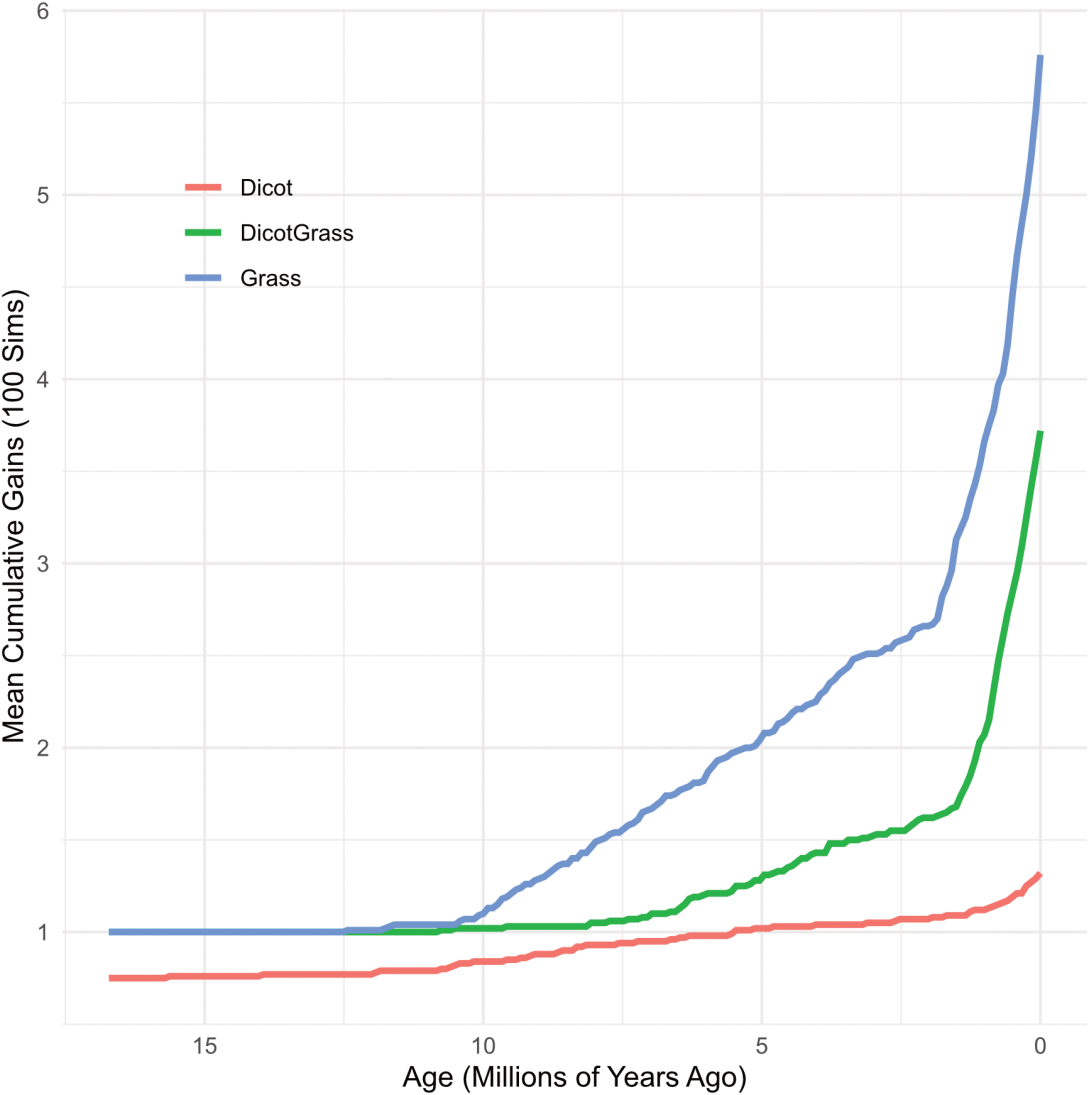
Temporal accumulation of feeding niche transitions. Accumulation through time was estimated via Stochastic Character Mapping (SCM) across 100 simulations. Curves represent the mean cumulative number of lineages transitioning into each state (cutting habits). While crown clades originated ~15 Ma, grass cutting (blue) transitions initiated at ~11 Ma, followed by a rapid, exponential increase in both grass and dicot-grass (green) states during the Plio-Pleistocene (<3 Ma). This pattern aligns with the global expansion of open grasslands in South America, contrasting with the stagnant accumulation of the dicot cutting (red).

Trait accumulation analysis shows that the evolution of cutting habits was characterized by a significant temporal lag after the initial diversification of clades (**Figure 3**). Although crown clades originated in the mid-Miocene, grass-cutting behavior did not emerge until ~10 Ma and remained at a steady rate until the late Pliocene. Subsequently, both grass and dicot-grass cutting states exhibited exponential increases in diversification rates during the Pleistocene (<2.5 Ma). This explosive radiation reveals that the diversification of grass cutting behavior did not occur immediately but instead reached its maximum evolutionary rate in tandem with the late-stage expansion of open grassy biomes. In contrast, dicot cutting lineages showed a stable accumulation pattern throughout the Neogene.

## 4 Discussion

Our study provides an integrated perspective on the evolutionary history of leaf-cutting ants. We achieved this by combining time-calibrated phylogenetic inference, ancestral state reconstruction, and ecological data within a phylogenetic comparative framework. First, we show that the origin and major diversification events of leaf-cutting ants coincide with Miocene–Pliocene environmental changes. This highlights the significant impact of large-scale climatic and geological processes on their evolution. Second, we also identify contrasting diversification dynamics among *Acromyrmex*, *Amoimyrmex*, and *Atta*. Third, our analyses demonstrate that cutting preferences did not evolve once but rather arose repeatedly in response to shifts in vegetation structure. Grass cutting emerged independently across several lineages.

### 4.1 Origin and diversification of leaf-cutting ants

Our divergence-time estimates date the origin of leaf-cutting ants to the early Miocene (~17–19 Ma), which is consistent with previous molecular dating studies based on phylogenomic and multilocus datasets (8, 25, 50). This time period coincided with a significant climate shift involving global warming and the growth of open, herbaceous, and shrubby vegetation, primarily in southern South America were forest areas gradually replaced by drier and more seasonal landscapes (56, 57). These landscape changes likely created ecological opportunities for large-scale herbivory and may have facilitated the early diversification of leaf-cutting ants.

The emergence of leaf-cutting ants during this period aligns with broader patterns in the evolution of fungus-growing ants. Branstetter et al. (50) emphasized that the colonization of arid and seasonally dry habitats by fungus growing ants was a critical step in the domestication of highly specialized fungal strains, which ultimately enabled large-scale herbivory. Our results support this idea, showing that the main radiation of leaf-cutting ants occurred after environmental conditions favorable to open habitats and increased plant turnover were established.

The major crown clades of leaf-cutting ants diversified during the late Miocene to early Pliocene (~10–5 Ma), a period marked by intensified aridification and climatic seasonality in South America. These changes were driven by large-scale geological and oceanographic processes, including the uplift of the Andes and the strengthening of the cold Humboldt Current, which was associated with Antarctic glaciation (58, 59). These events reshaped precipitation regimes and promoted the expansion of xerophytic vegetation (57), which likely facilitated the southward dispersal and regional diversification of leaf-cutting ants. The concentration of divergence events within this timeframe indicates that climatic factors played a pivotal role in shaping present-day biodiversity.

### 4.2 Phylogenetic relationships of leaf cutting ants

Leaf-cutting ants exhibit pronounced morphological, ecological, and behavioral diversity. This diversity has historically led to the description of numerous subspecies and infrageneric groups (23, 60). However, the lack of a thorough, contemporary taxonomic review, combined with largely regional studies, has complicated the interpretation of evolutionary relationships (61). Our study integrates molecular phylogenetic inference, diagnostic morphology, and geographic information to provide a robust framework for evaluating these relationships in a temporal context.

Our analyses indicate that *Acromyrmex* origin (~13 Ma) is consistent with previous phylogenetic estimates reported by Schultz and Brady (8)Branstetter et al. (50), and Micolino et al. (25). The late Miocene-early Pliocene origin of grass cutting within the *Acromyrmex* genus suggests that this adaptation emerged in response to the increased availability of grasses in open, seasonally dry environments rather than representing an ancestral trait. This pattern aligns with ancestral state reconstructions that indicate dicot cutting as the ancestral condition and multiple independent origins of grass cutting. Relatively deep internal divergences among *Acromyrmex* clades imply a more protracted and heterogeneous diversification history than that observed in *Atta*. Our results partially align with earlier morphological hypotheses regarding the infrageneric structure of *Acromyrmex* (24), especially the close relationship between *A. heyeri* and *A. lundii*. However, the questionable monophyly of traditional subgeneric divisions such as Moellerius highlights the need for a revised classification based on phylogenomic evidence.

In contrast, *Atta* exhibits a younger and faster radiation. Its crown age is estimated at ~5 Ma, with four major clades arising within a narrow temporal window during the Pliocene. The short internal branches and overlapping divergence-time estimates among these clades suggest a rapid diversification process driven by ecological expansion into new niches rather than long-term persistence. This pattern contrasts with that of *Acromyrmex*, suggesting that, although the two genera currently occupy similar ecological niches, they evolved under different temporal and environmental contexts. Several studies indicate a much more recent diversification in *Atta*. Phylogenomic analyses by Barrera et al. (62) revealed that the genus is quite recent, with extant species dating back to the early Pleistocene (~1.8-0.3 million years ago), suggesting a rapid and recent radiation. Consistent with this view, Micolino et al. (25) reported similar divergence time estimates for *Atta*, supporting the idea that *Atta* is a relatively recent radiation among leaf-cutting ants.

The phylogenetic position of the *Acromyrmex* only grass-cutter clade species has implications for the status of the subgenus *Moellerius*. According to Forel’s (21) original concept, centered on *A. landoltii* and *A. balzani*, this clade corresponds closely to the *Moellerius* subgenus. However, (22) expanded this definition by including species like *A. heyeri* based on mandibular and metasomal traits, which is inconsistent with the presented phylogenetic evidence (**Figure 2**). *A. heyeri* is a notable example of a grass-cutting species embedded within a predominantly dicot-cutting clade because it is classified as the sister species of *A. lundii*. This pattern suggests an independent and recent shift in cutting preference. The close phylogenetic relationship and overlapping geographic distributions of *A. heyeri*, *A. lundii*, and *A. ambiguus* suggest that local ecological conditions may have promoted rapid behavioral divergence. In regions where open grasslands and mixed habitats coexist, competition may intensify among closely related species with similar niches (63). Under these conditions, adopting a grass-based foraging strategy could reduce competitive overlap by exploiting less-contested resources and facilitate ecological differentiation. Thus, the derived grass-cutting behavior of *A. heyeri* may be a localized adaptive response rather than a deep phylogenetic constraint.

Most *Acromyrmex* species are clearly specialized for either dicot or grass cutting, with one notable exception: *A. lobicornis*. This species has been consistently documented exploiting both vegetation types, representing the only known dicot-grass cutter within the genus (64, 65). Its intermediate strategy may reflect a more generalist foraging ecology or a transitional state in the evolution of cutting preferences. *A. lobicornis* broad ecological tolerance and ability to occupy open and semi-open habitats may reduce selective pressures favoring strict specialization (66). This illustrates that, while cutting preferences are phylogenetically informative, they are not evolutionarily fixed and may shift in response to local environmental conditions.

In contrast to *Acromyrmex*, *Atta* cutting preferences show no clear phylogenetic structure. Grass cutters and dicot-grass cutters are distributed across multiple clades (**Figure 2B**). Ancestral nodes retain strong support for dicot cutting. These results suggest that grass-cutting behavior evolved independently and repeatedly within *Atta* rather than originating from a single specialized clade. The absence of a distinct grass-cutter clade within *Atta* suggests that transitions toward exploiting grassy substrates occurred in response to local ecological pressures and were potentially facilitated by behavioral flexibility or shared morphological traits enabling repeated shifts when conditions favor them (e.g., 67).

In our analyses, *Amoimyrmex* was found as a sister of *Acromyrmex* + *Atta*, though the posterior support was low. This result aligns with some previous phylogenetic studies (25, 50). This phylogenetic placement and evolutionary history of *Amoimyrmex* shed light on the early diversification of leaf-cutting ants. *Amoimyrmex* is a recently described genus that includes the former *Acromyrmex striatus* and is strongly associated with grass cutting and open habitats (68). This makes its phylogenetic position particularly informative for understanding the origins of cutting specialization within the leaf-cutting ants (**Figure 2**). Its inferred early divergence suggests that grass cutting in this genus represents an independent evolutionary trajectory, distinct from the only grass-cutter clade identified within *Acromyrmex*, the independent origin of grass cutting in *A. heyeri*, and as well as the multiple independent origins identified within *Atta*. This reinforces the broader pattern of ancestral-state reconstruction, which indicates that grass-cutting behaviour evolved repeatedly under similar ecological pressures.

### 4.3 Evolutionary origins of cutting preferences

Ancestral state reconstruction revealed a clear and consistent pattern indicating that grass-cutting behavior evolved independently multiple times within leaf-cutting ants rather than arising once and diversifying through shared ancestry. Dicot cutting was strongly supported as the ancestral condition across deep nodes in the phylogeny. In contrast, grass cutting and dicot-grass cutting were reconstructed as derived states. This pattern is particularly evident within *Acromyrmex*, where most grass-cutting species form a well-supported monophyletic clade (**Figure 2**), with the notable exception of *A. heyeri*.

The estimated divergence time for the *Acromyrmex* only grass-cutter clade species is ~4 million years ago (Ma), placing its origin in the late Miocene–early Pliocene. This period was characterized by increasing climatic seasonality in northeastern Argentina and southern Brazil. During this time, grassy savannas expanded and C_4_ plants diversified throughout the region (69, 70). Our trait accumulation analysis shows that grass-cutting behavior did not diversify immediately after the origin of crown clades. Rather, diversification began around 10 Ma and reached its maximum evolutionary rate during the Pleistocene, likely tracking the late-stage expansion of open, grassy biomes. The close temporal correspondence between these environmental changes and the emergence of a grass-specialized clade suggests that shifts in vegetation structure likely provided new ecological opportunities that favored the evolution of grass-cutting behavior. More broadly, the repeated appearance of grass cutting across the phylogeny indicates that similar selective pressures operated on different lineages at different times and regions.

Large-scale geological and climatic processes in South America provide a plausible backdrop for these evolutionary transitions. Repeated marine incursions, including the Paraná/Entrerriense transgressions, inundated large areas of the Chaco–Paraná lowlands during the middle Miocene (~15–13 Ma) and again during the late Miocene (~10–5 Ma). These events substantially altered lowland habitats, creating biogeographic barriers and changing dispersal routes (71, 72). Such fragmentation and reconnection of habitats likely influenced population isolation and secondary contact, setting the stage for lineage divergence and ecological differentiation. On a continental scale, Neogene tectonic processes, particularly Andean uplift, reorganized drainage systems, altered precipitation regimes, and reshaped South American landscapes (73). These changes generated a heterogeneous mosaic of wet forests, savannas, and drylands, widely recognized as a driver of diversification through vicariance and ecological opportunity. Within this dynamic environmental context, the timing of leaf-cutting ants diversification and the emergence of grass-cutting lineages can be interpreted as responses to a rapidly changing landscape. Additionally, the scarcity of extensive tropical grasslands has been suggested as a critical factor in the decline of true leaf-cutting ant taxonomic diversity toward the equator, connecting diversification patterns with the distribution of open habitats (74). The present-day distributions of several taxa also align with the inferred Pleistocene vegetational dynamics. This suggests that cycles of grassland expansion and contraction contributed to the isolation and diversification of grass-cutting forms (74).

Climatic trends during the late Miocene further reinforced these effects. Global cooling, declining atmospheric CO_2_ concentrations, and increasing climatic seasonality promoted the worldwide expansion of open, seasonal habitats (75). During the late Miocene-early Pliocene, C_4_ grasses expanded geographically and ecologically, transforming plant communities and favoring grass-dominated ecosystems (70, 76). Marine transgressions, Andean uplift, late Miocene cooling, and grassland expansion constrained dispersal while creating new selective environments (73). These processes likely promoted repeated ecological shifts, including the independent evolution of grass-cutting behavior within leaf-cutting ants.

Strong support for dicot cutting as the ancestral foraging mode implies that the transition to grass cutting required overcoming substantial mechanical and ecological challenges. Grasses typically have higher silica content, greater tissue toughness, and different growth patterns than dicotyledonous plants (18, 37). These characteristics impose functional constraints on herbivores. The convergent evolution of grass-cutting strategies in both leaf-cutting ants suggests that environmental changes repeatedly imposed selective pressures favoring specialization on grassy substrates rather than a single evolutionary innovation giving rise to all grass-cutting lineages. Ecological data from Paraguay indicate that grass-cutting (e.g., *A. balzani* and *A. fracticornis*) are confined to open rangelands with low structural complexity. In contrast, dicot-cutting (e.g., *Atta laevigata* and *A. sexdens)* inhabit woodlands and mixed habitats, reflecting a broader niche breadth (77). Their overall abundance increases as habitat complexity decreases, indicating ecological differentiation along vegetation gradients (77). These repeated transitions are consistent with known patterns of morphological specialization. Grass-cutting species tend to have shorter, more robust mandibles that can withstand abrasion from silica-rich grasses (37, 78). In contrast, dicot cutters usually have longer, slimmer mandibles adapted to softer foliage (79). Mandibular wear is a significant selective force (80), so these functional differences provide a mechanistic basis for the recurrent evolution of grass specialization observed across the phylogeny (**Figure 2**).

Cutting strategies may reflect ecological filtering under tropical constraints. According to Fowler (74), the increasing concentrations of fungicidal or fungistatic allelochemicals in tropical regions, coupled with the high complexity of vegetation, may have limited the performance of fungi and reduced the foraging efficiency of leaf-cutting ants in tropical forests. This challenges the idea that leaf-cutting ants originated strictly in forests. Within this framework, grass, dicot-grass, and dicot cutting strategies can be interpreted as alternative ecological solutions along gradients of plant chemistry and habitat structure. Dicot specialists, which are predominantly associated with Clade-A fungi, may be better suited to chemically diverse forest environments. In contrast, grass and mixed cutters, which are more frequently linked to Clade-B cultivars, are often associated with open or structurally simpler habitats (81). However, the weak genetic differentiation among Clade-A fungi and the sharing of identical clones between sympatric specialists suggest that fungal lineages are not strictly partitioned by cutting strategy. This indicates that associations are more flexible and shaped by ecological context than by strict co-diversification.

### 4.4 Broader implications and concluding remarks

Together, our results demonstrate that the cutting preferences of leaf-cutting ants are best explained by repeated adaptive responses to significant environmental changes rather than by a single evolutionary origin. Major geological and climatic processes during the Miocene–Pliocene epoch generated a heterogeneous and dynamic landscape in South America, repeatedly opening ecological opportunities associated with the expansion of open, grass-dominated habitats.

The contrasting diversification patterns of *Acromyrmex*, *Amoimyrmex*, and *Atta* show that similar ecological roles can evolve through different pathways influenced by timing, lineage structure, and ecological opportunity. By integrating phylogenetic, temporal, and ecological evidence, our study provides a robust framework for understanding how major environmental transformations during the Miocene shaped the diversification and ecological dominance of leaf-cutting ants. Within this framework, grass-cutting specialization emerges as a repeatedly evolved adaptive response to environmental change rather than as a single conserved innovation.

## Data Availability

The datasets presented in this study can be found in online repositories. The names of the repository/repositories and accession number(s) can be found in the article/**Supplementary Material**.
