# Supplementary material for: Phylogenetic insights into the diversification of cutting strategies in leaf-cutting ants
Source: Front Insect Sci. 2026 Apr 7;6:1778418. doi: 10.3389/finsc.2026.1778418 (PMC13095832; doi:10.3389/finsc.2026.1778418)
Supplement: Supplementary file 1 [file Table1.docx]

***Supplementary Material***

# PCR protocol and primers

PCR reactions for COI-tRNAleu-COII and LWRh were performed in a 50 μL reaction volume containing: 1 μL genomic DNA extract (50-100 ng), 5 μL 10x buffer, 0.2 μL Taq polymerase, 3 μL MgCl2, 5 μL dNTPS, 1 μL of each primer (10 μM), and 33.8 μL distilled water. PCR reactions for EFαF1; EFαF2 and Wg genes were performed in a 20 μL reaction volume containing: 4 μL genomic DNA extract (50-100 ng), 0.5 μL REDExtract-N-Amp PCR ReadyMix, 0.5 μL of each primer (10 μM) and 5 μL distilled water. Thermal cycling conditions in both cases were as follows: denaturation at 95°C for 3 minutes, followed by 37 cycles of denaturation at 94°C for 1 minute, quenching at 50°C for 1 minute (or 51°C, depending on the fragment to be amplified), and extension at 74°C for 1 minute, followed by a final extension at 74°C for 10 minutes. PCR products were visualized on a 1% agarose gel stained with GelRed (Biotium, Hayward, CA, USA) and enzyme purified using 0.5 µL (10u) of FastAP (thermosensitive alkaline phosphatase) and 1 µL (1u) of ExoI (exonuclease I), incubated at 37°C for 15 min and stopped at 85°C for 15 min.

**Table S1**. Primer names, location and sequence used to amplify each of the used fragments.

| **Primer Name** | **Location** | **Dir.** | **Sequence (5' - 3')** | **Reference** |
| --- | --- | --- | --- | --- |
| C1-J-2828 | Mitochondrial[(Solomon, 2007)](https://www.zotero.org/google-docs/?yfNJLG) | F | ATTCATTCTTATCTTGAAATATTATTTC | [Solomon, (2007)](https://www.zotero.org/google-docs/?he1dG9) |
| C2-N-3386 | Mitochondrial | R | TTCATAAGTTCAGTATCATTGGTG |  |
| F1-1424F | Nuclear | F | GCGCCKGCGGCTCTCACCACCGAGG | [Schultz and Brady, (2008)](https://www.zotero.org/google-docs/?vJNir0) |
| F1-1829R | Nuclear | R | GGAAGGCCTCGACGCACATMGG |  |
| F2-557F | Nuclear | F | GAACGTGAACGTGGTATYACSAT | [Schultz and Brady, (2008)](https://www.zotero.org/google-docs/?XIP7bj) |
| F2-1118R | Nuclear | R | TTACCTGAAGGGGAAGACGRAG |  |
| LR143F | Nuclear | F | GACAAAGTKCCACCRGARATGCT | [Ward and Downie, (2005)](https://www.zotero.org/google-docs/?YXty33) |
| LR639ER | Nuclear | R | YTTACCGRTTCCATCCRAACA |  |
| Wg578F | Nuclear | F | TGCACNGTGAARACYTGCTGGATGCG | [Ward and Downie, (2005)](https://www.zotero.org/google-docs/?OYgQNj) |
| Wg1032R | Nuclear | R | ACYTCGCAGCACCARTGGAA |  |

**Table S2**. Fossils used for the calibration and age estimation using the "fossilized birth-death process" (FBD) model.

| **Fossil** | **Formation** | **Age ranges (Ma)** | **References** |
| --- | --- | --- | --- |
| †Acanthognathus poinar  Baroni Urbani 1994 | Dominican amber | 20,43–13,65 | [(Baroni Urbani and de Andrade, 1994; Bolton and Fisher, 2012)](https://www.zotero.org/google-docs/?F4L2PN) |
| †Apterostigma electropilosum  Schultz 2007 | Dominican amber | 20,43–13,65 | [(Schultz, 2007; Bolton and Fisher, 2012)](https://www.zotero.org/google-docs/?WF1CWr) |
| †Cephalotes integerrimus  Vierbergen & Scheven 1995 | Dominican amber | 20,43–13,65 | [(Vierbergen and Scheven, 1995; De Andrade and Baroni Urbani, 1999)](https://www.zotero.org/google-docs/?zUlyxW) |
| †Cyphomyrmex taino  de Andrade 2003 | Dominican amber | 20,43–13,65 | [(De Andrade, 2003)](https://www.zotero.org/google-docs/?JYm1cz) |
| †Pheidole tertiaria  Carpenter 1930 | Florissant formation | 37,2–33,9 | [(Carpenter, 1930; Casadei-Ferreira et al., 2019)](https://www.zotero.org/google-docs/?zW7YsE) |
| †Trachymyrmex primaevus  Baroni Urbani 1980 | Dominican amber | 20,43–13,65 | [(Baroni Urbani, 1980; Bolton and Fisher, 2012)](https://www.zotero.org/google-docs/?ojp32u) |

# Leaf-cutting occurrence dataset

The occurrence dataset was compiled from records of all leaf-cutting ant species gathered from multiple sources. First, we used records from our own field surveys. Second, we used records reported in peer-reviewed publications and relevant technical reports. These records were cross-checked against those curated in the Global Ant Biodiversity Informatics (GABI) repository. The GABI repository is currently accessible through [AntMaps.org](http://antmaps.org) [(Guénard et al., 2017)](https://www.zotero.org/google-docs/?4HTkSF). Third, we obtained additional occurrence data from digital biodiversity databases, particularly the Global Biodiversity Information Facility [(GBIF, 2024b, 2024a)](https://www.zotero.org/google-docs/?053Yzk). Finally, we included records from [AntWeb.org](http://antweb.org) [(AntWeb, 2024)](https://www.zotero.org/google-docs/?f8JgfO), a repository curated by specialists that provides an extensive taxonomic catalog, specimen records mainly from biological collections, photographs, and natural history information for ants. After cleaning the dataset, we kept only the records with coordinates (~43000 occurrence records). Then, following [Dinerstein et al., (2017)](https://www.zotero.org/google-docs/?SiWCWZ), we assigned biome information to each record. Next, we summarized the data by counting the number of occurrences for each combination of genus, species, and biome. Next, we calculated the proportional contribution of each record within a species. To do so, we grouped the data by genus and species. Then, we divided the number of occurrences in each biome by the total number of occurrences for that species. This resulted in a proportional metric representing the relative frequency of each species across biomes.

# References

[AntWeb (2024). AntWeb Version 8.103.2. California Academy of Science. Available at: https://www.antweb.org](https://www.zotero.org/google-docs/?0o6doF)

[Baroni Urbani, C. (1980). First description of fossil gardening ants. (Amber Collection Stuttgart and Natural History Museum Basel; Hymenoptera: Formicidae. I: Attini). *Stuttg. Beitr. Naturkd. Ser. B (Geol. Palaontol.)* 54, 1–13. doi: 10.5281/zenodo.26798](https://www.zotero.org/google-docs/?0o6doF)

[Baroni Urbani, C., and de Andrade, M. L. (1994). First description of fossil Dacetini ants with a critical analysis of the current classification of the tribe. *Stuttgarter Beiträge zur Naturkunde* 198, 1972–2007.](https://www.zotero.org/google-docs/?0o6doF)

[Bolton, B., and Fisher, B. L. (2012). *Taxonomy of the cerapachyine ant genera Simopone Forel, Vicinopone gen. n. and Tanipone gen. n. (Hymenoptera: Formicidae)*. doi: www.mapress.com/zootaxa/](https://www.zotero.org/google-docs/?0o6doF)

[Carpenter, F. M. (1930). The fossil ants of North America. *Bull. Mus. Comp. Zool.* 70(1), 1–66.](https://www.zotero.org/google-docs/?0o6doF)

[Casadei-Ferreira, A., Chaul, J. C. M. M., and Feitosa, R. M. (2019). A new species of Pheidole (Formicidae, myrmicinae) from dominican amber with a review of the fossil records for the genus. *ZooKeys* 2019, 117–125. doi: 10.3897/zookeys.866.35756](https://www.zotero.org/google-docs/?0o6doF)

[De Andrade, M. L. (2003). First descriptions of two new amber species of Cyphomyrmex from Mexico and the Dominican Republic (Hymenoptera: Formicidae). *Beitr. Entomol.* 53(1), 131–139. doi: 10.21248/contrib.entomol.53.1.131-139](https://www.zotero.org/google-docs/?0o6doF)

[De Andrade, M. L., and Baroni Urbani, C. (1999). Diversity and adaptation in the ant genus Cephalotes, past and present. *Stuttgarter Beiträge zur Naturkunde. Serie B. Geologie und Paläontologie* Ser. B, 1–889.](https://www.zotero.org/google-docs/?0o6doF)

[Dinerstein, E., Olson, D., Joshi, A., Vynne, C., Burgess, N. D., Wikramanayake, E., et al. (2017). An Ecoregion-Based Approach to Protecting Half the Terrestrial Realm. *BioScience* 67, 534–545. doi: 10.1093/biosci/bix014](https://www.zotero.org/google-docs/?0o6doF)

[GBIF (2024a). Acromyrmex, GBIF Occurrence Download. doi: https://doi.org/10.15468/dl.a69e3r](https://www.zotero.org/google-docs/?0o6doF)

[GBIF (2024b). Atta, GBIF Occurrence Download. doi: https://doi.org/10.15468/dl.tk8vff](https://www.zotero.org/google-docs/?0o6doF)

[Guénard, B., Weiser, M. D., Gómez, K., Narula, N., and Economo, E. P. (2017). The Global Ant Biodiversity Informatics (GABI) database: Synthesizing data on the geographic distribution of ant species (Hymenoptera: Formicidae). *Myrmecological News* 24, 83–89. doi: https://doi.org/10.25849/myrmecol.news_024:083](https://www.zotero.org/google-docs/?0o6doF)

[Schultz, T. R. (2007). The fungus-growing ant genus Apterostigma in Dominican amber. *Memoirs of the American Entomological Institute* 80, 425–436.](https://www.zotero.org/google-docs/?0o6doF)

[Schultz, T. R., and Brady, S. G. (2008). Major evolutionary transitions in ant agriculture. *Proceedings of the National Academy of Sciences of the United States of America* 105, 5435–5440. doi: 10.1073/pnas.0711024105](https://www.zotero.org/google-docs/?0o6doF)

[Solomon, S. E. (2007). Biogeography and Evolution of Widespread Leafcutting Ants, *Atta* Spp. (Formicidae, Attini). The University of Texas at Austin.](https://www.zotero.org/google-docs/?0o6doF)

[Vierbergen, G., and Scheven, J. (1995). Nine new species and a new genus of Dominican amber ants of the tribe (Cephalotini Hymenoptera: Formicidae). *Creation Research Society Quarterly* 32, 158–170.](https://www.zotero.org/google-docs/?0o6doF)

[Ward, P. S., and Downie, D. A. (2005). The ant subfamily Pseudomyrmecinae (Hymenoptera: Formicidae): Phylogeny and evolution of big-eyed arboreal ants. *Systematic Entomology* 30, 310–335. doi: 10.1111/j.1365-3113.2004.00281.x](https://www.zotero.org/google-docs/?0o6doF)
